# Characteristics of prostate cancer detection rate (PCDR) in Chinese Han population under different prostate biopsy methods

**DOI:** 10.18632/oncotarget.16512

**Published:** 2017-03-23

**Authors:** Yongsheng Pan, Bianjiang Liu, Yuan Huang, Jun Wang, Xiao Li, Cheng Zhang, Jie Wu, Yuxiao Zheng, Chao Qin, Gong Cheng, Lixin Hua, Zengjun Wang

**Affiliations:** ^1^ Department of Urology, The First Affiliated Hospital of Nanjing Medical University, Nanjing 210029, China

**Keywords:** prostate cancer, biopsy, Chinese Han population, diagnosis

## Abstract

We analyzed the improvement of prostate cancer detection rate (PCDR) in Chinese Han population and summarized the characteristics of prostate cancer (PCa) with the advancement of prostate biopsy technologies. From March 1999 to March 2015, 3762 patients underwent the systematic 6-, 8- or 13-core biopsy, guided by finger or transrectal ultrasound (TRUS) at our center. The PCDR under different PSA intervals and different biopsy methods were analyzed. The trends of PSA level, age and Gleason score of PCa patients were summarized. The PCDR of finger-guided 6- and 8-core biopsies were 30.8% (340/1103) and 36.7% (147/401) respectively. In 2258 patients with TRUS-guided 13-core biopsies, 992 (43.9%) were diagnosed as PCa, higher than that with finger-guided biopsies (43.9% vs. 32.4%, *p* < 0.001). The PCDR of prostate peripheral zone was higher than that of medial zone (37.5% vs. 31.4%, *p* < 0.001). Interestingly, the PCDR of extra 13th core was higher than the mean positive rate of other 12 cores (70.7% vs. 56.0%, *p* < 0.001). The systematic 13-core prostate biopsy guided by TRUS is safe, effective, and economic for PCa diagnosis in developing countries like China. The extra 13th core biopsy is beneficial to increase the PCDR.

## INTRODUCTION

Prostate cancer (PCa) is one of the most common malignant tumors and the second leading cause of cancer death in European and American population [1, 2]. Transperineal or transrectal prostate biopsy is the gold standard for PCa diagnosis. Hodge *et al*. reported firstly the systematic sextant biopsy protocol under transrectal ultrasound (TRUS) in 1989 [3]. Since then, extended prostate biopsy scheme with more biopsy cores was performed to increase the prostate cancer detection rate (PCDR) [4–6]. The guidelines of the European and American Urological Associations have recommended the systematic 10-12-core TRUS-guided prostate biopsy as the gold standard for primary diagnosis [7, 8]. Currently, the systematic 12-core prostate biopsy guided by TRUS is performed in most medical centers.

Asia population, especially Chinese Han population, has significantly lower incidence of PCa, but higher proportion of locally advanced and metastatic diseases than that of western population [9]. The differences of PSA screening efforts and prostate biopsy technologies lead to the disparity. However, the incidence of PCa in China grows faster than European and American countries with the development of economy and society. The epidemiological and pathogenetic characteristics of PCa in Chinese Han population, especially the transmutation in decades, remain unclear. Our study analyzed the 16-year data and summarized the PCDR in Chinese Han population under different prostate biopsy methods at our center.

## RESULTS

Of the total 3762 patients underwent the transrectal prostate biopsy, the PCDR was 39.3% (1479/3762). The other 2283 cases were normal prostate tissues, prostatitis, or benign prostate hyperplasia. 3588 cases had complete clinical data including total PSA (tPSA), free PSA (fPSA), age, PV, DRE, and TRUS results. The PCDR in patients with different PSA levels, ages, prostate volumes, f/t PSA ratio, PSA density (PSAD), DRE and TRUS results were shown in Table 1. The higher tPSA value, older age, smaller prostate size, lower f/t ratio, and higher PSAD correlated with higher PCDR (*p* < 0.001). The PCDR of patients with abnormal DRE was 57.6% (951/1652), higher than that of patients with an elevated tPSA level (range, 4–160 ng/ml) (37.8%; 1283/3398). The ratio of patients with Gleason score ≥ 8 in abnormal DRE group was higher than that of elevated tPSA group (35.1% vs. 25.4%, *p* < 0.001).

**Table 1:** The PCDR in patients with different PSA levels, PV, ages, DRE results and hypoechoic results

Variable	Positive, *n* (%)	Negative, *n* (%)	*p* value
PSA (ng/ml)			< 0.001
0–4	26 (13.7)	164 (86.3)	
4.01–10	254 (21.4)	931 (78.6)	
10.01–20	340 (30.2)	784 (69.8)	
20.01–50	378 (53.2)	333 (46.8)	
50.01–100	231 (79.4)	60 (20.6)	
> 100	250 (95.8)	11 (4.2)	
PV (cm^3^)			< 0.001
< 40	619 (46.4)	714 (53.6)	
40–60	406 (40.4)	598 (59.6)	
60–80	217 (33.9)	424 (66.1)	
> 80	237 (30.2)	547 (69.8)	
Age (years)			< 0.001
< 50	11 (12.8)	75 (87.2)	
51–60	119 (27.4)	316 (72.6)	
61–70	502 (34.8)	942 (65.2)	
71–80	731 (45.6)	872 (54.4)	
> 80	116 (59.8)	78 (40.2)	
DRE results			< 0.001
Positive	951 (57.6)	701 (42.4)	
Negative	528 (25.0)	1 582 (75.0)	
Hypoechoic			< 0.001
Positive	743 (57.9)	540 (42.1)	
Negative	736 (29.7)	1 743 (70.3)	

From March 1999 to December 2006, 1103 patients underwent finger-guided systematic sextant biopsies. The PCDR was 30.8% (340/1103). From January 2007 to December 2008, the extended finger-guided 8-core biopsy was performed on 401 patients. The PCDR was 36.7% (147/401), higher than that of sextant biopsy (χ^2^ = 4.570, *p* = 0.033). Since January 2009, 2258 patients underwent TRUS-guided 13-core biopsy. The PCDR was 43.9% (992/2258), significantly higher than that of finger-guided 8-core biopsy (χ^2^ = 7.359, *p* = 0.007). Further analysis revealed that the PCDR of TRUS-guided biopsy (43.9%, 992/2258) was higher than that of finger-guided biopsy (32.4%, 487/1504; χ^2^ = 50.5, *p* < 0.001). However, TRUS-guided biopsy did not show higher detection rate of high grade PCa (29.6%, 294/992) than two finger-guided biopsies (28.9% in sextant biopsy, 99/340; 32% in 8-core biopsy, 47/147). The PCDR of TRUS-guided 13-core biopsy was close to that of systematic 12-core biopsy (43.1%, 973/2258; χ^2^ = 0.325, *p* = 0.568). However, the positive rate of the 13th core was 70.7%, significantly higher than that of the mean positive rate of the systematic 12-core in patients with confirmed PCa (56.0%; χ^2^ = 51.6, *p* < 0.001). The PCDR of lateral biopsy was higher than that of medial biopsies (37.5% vs 31.4%; χ^2^ = 18.1, *p* < 0.001).

The biopsy-related complications occurred in 1562 (41.5%) patients (Table 2). Among 1348 cases of minor complications, 1312 patients had slight gross hematuria or microscopic hematuria. 31 patients had hemospermia. Other 5 cases had the transient sexual dysfunction. 214 patients suffered severe complications, such as rectal bleeding (72 cases), gross hematuria (56 cases, 14 cases with acute urinary retention), fever more than 39–40°C (63 cases, 1 case with septic shock), vasovagal response (21 cases), hepatorenal function failure (1 case), and death (1 case, due to cardiovascular accident).

**Table 2:** The biopsy-related complications

Complications	Number (%)
Minor complications	1348
Slight gross hematuria or microscopic hematuria	1312 (34.9%)
Hemospermia	31 (0.8%)
Sexual dysfunction	5 (0.1%)
Severe complications	214
Rectal bleeding	72 (1.9%)
Gross hematuria	56 (1.5%)
Fever	63 (1.7%)
Vasovagal response	21 (0.6%)
Hepatorenal function failure	1 (0.03%)
Death	1 (0.03%)

**Figure 1:**
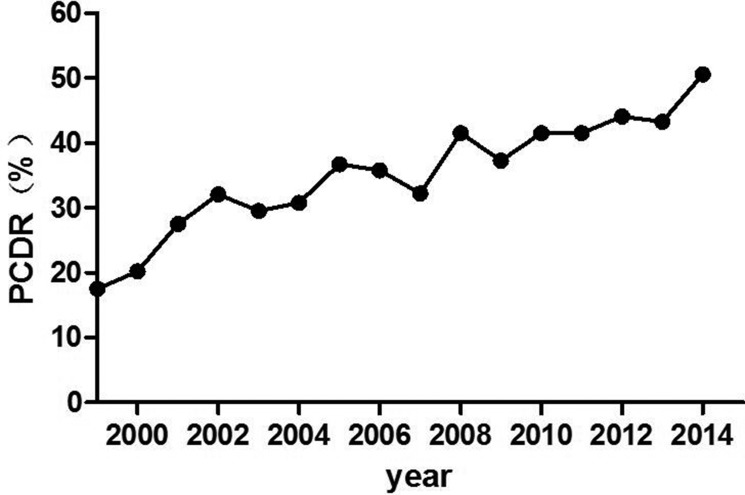
Trend of the PCDR over the years The PCDR increased from 17.5% in 1999 to 50.6% in 2015.

**Figure 2:**
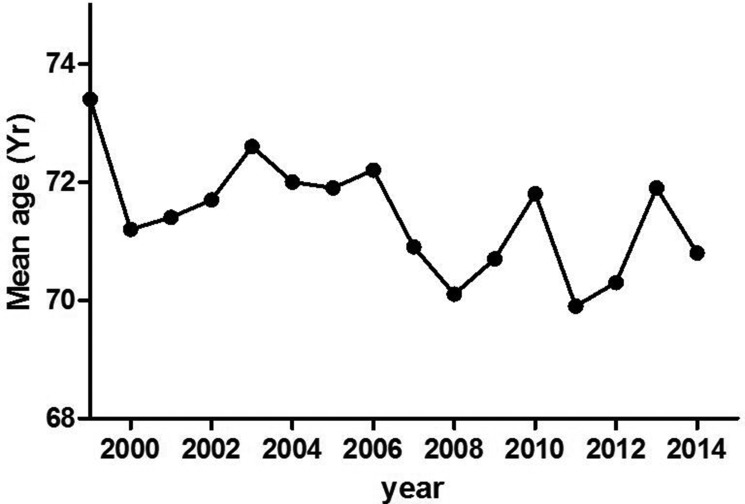
Trend of mean age of PCa patients detected by prostate biopsy over the years The mean age decreased from 73.4 years old in 1999 to 70.8 years old.

**Figure 3:**
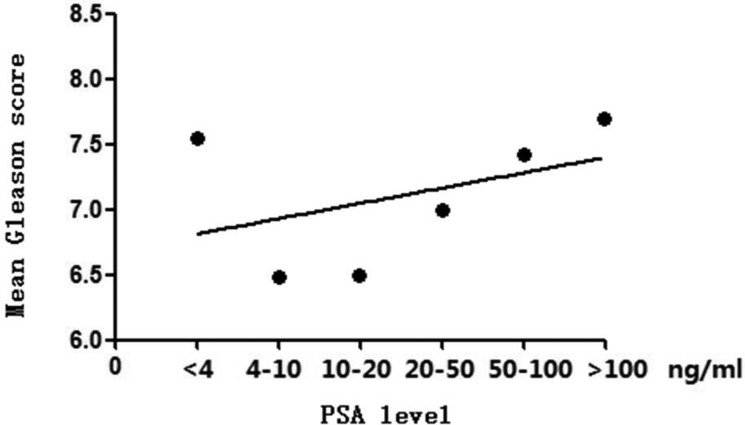
Trend of mean Gleason score with the increase of PSA levels The mean Gleason score increased with PSA levels. The mean Gleason score of PSA level lower than 4.0 ng/ml was higher than that of PSA intervals 4.0 to 10, 10 to 20, 20 to 50 ng/ml.

From 1999 to 2015, the PCDR increased from 17.5% to 50.6% (Figure 1). The mean age of patients diagnosed with PCa decreased from 73.4 years to 70.8 years (Figure 2). As PSA level increased, the Gleason score of patients with PCa increased gradually. However, the mean Gleason score of patients with PSA level lower than 4.0 ng/ml was higher than that of PSA intervals 4.0 to 10, 10 to 20, 20 to 50 ng/ml (Figure 3). After 2009, 885 patients with elevated tPSA level and 1236 patients with lower urinary tract symptoms (LUTS) underwent prostate biopsy respectively. The PCDR between two groups is no obvious difference (45.2%, 400/885 vs 43.2%, 534/1236). However, the ratio of patients with Gleason score ≥ 8 in LUTS group was higher than that of evaluated tPSA group (16.7% vs 36.9%, *p* < 0.001).

**Figure 4:**
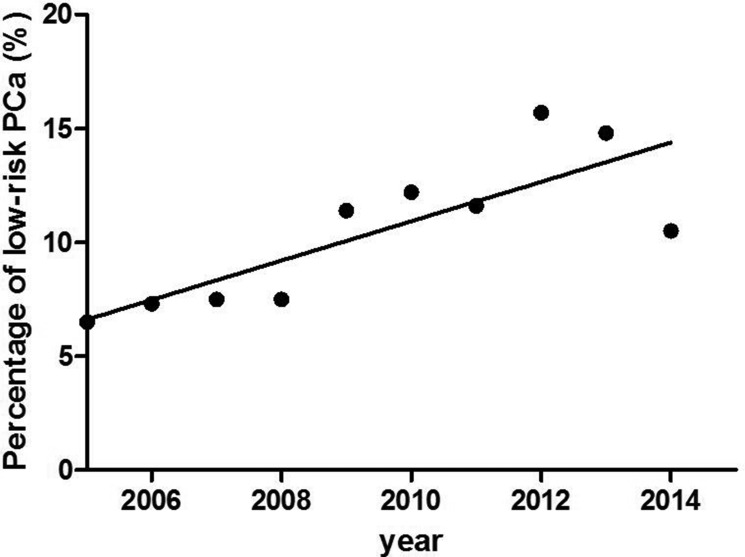
The percentage of low-risk PCa There was a significant increase in the percentage of low-risk PCa from 2005 to 2015.

Due to the popular application of PSA test in China since 2009, more and more clinically insignificant PCa was detected occasionally. According to NCCN standard, the tumor with simultaneous Gleason score ≤ 6, PSA < 10 ng/ml and TNM stage ≤ T2aN_0_M_0_ was clinically insignificant [10]. PCa with no clinical significance increased from 6.5% to 15.7% in this group (Figure 4).

## DISCUSSION

TRUS-guided systematic multiple cores biopsy has been widely adopted in most advanced medical centers in China. The TRUS-guided biopsy could increased PCDR significantly compared with the finger-guided biopsy. However, the optimal core number and distribution remain controversial. The false-negative rate of sextant scheme reached 41%, while an extended 10-core scheme could improve PCDR relatively [4, 7]. Elabbady *et al*. confirmed that the 12-core biopsy increased PCDR from 25.8% to 36.4% compared with 6-core biopsy [11]. Our current study showed that the PCDR improved from 30.8% to 36.7%, and finally to 43.9% with the change of biopsy cores from 6 to 8 and 13. The result was similar with data of other countries [4, 7, 11]. Further analysis demonstrated that the positive rate of the extra 13th core was significantly higher than the mean positive rate of systematic 12-core biopsy. Additionally, our study suggested that the ratio of patients with Gleason score ≥ 8 in abnormal DRE group was higher than that of evaluated tPSA group (range, 4–160 ng/ml). It meant that patients with abnormal DRE had a higher degree of malignancy.

Presti *et al*. performed 10-core systematic biopsy on 483 patients with lateral biopsy of the peripheral zone at the base and mid gland added to routine sextant biopsy regimen [12]. They found that tumor was predominantly located in the peripheral zone. Our data also showed a higher detection rate of the peripheral zone in the comparison of middle zone. The result was consistent with the literature and our previous work [12, 13].

Further, PCa seems predominantly to be located in the apical region of peripheral zone. Breslow *et al*. found that the majority of carcinomas occurred within 5–15 mm of the apical region of the gland [14]. Bittner *et al*. revealed that the most common tumor detection site was the anterior apex [15]. Considering transperineal approach has the advantage of the detection of tumors at the anterior or apical prostate, the anterior apex regions might potentially be the “under sampled region” of transrectal prostate biopsy [15, 16]. However, our study reported the 39.3% PCDR, higher than that of study by Mai *et al*. (35.5%) [17]. The difference was possibly originated from different inclusion criteria. Patients with PSA levels greater than 100 ng/ml were included by our study but excluded by Mai *et al*. Our data showed that the PCDR of transrectal approach was close to that of transperineal biopsy in Chinese Han population, which was different from the studies by Vis *et al*. and Emiliozzi *et al*. [18, 19].

The biopsy-related complications in Chinese Han population were similar with western population. Rectal bleeding, gross hematuria and fever were considered as severe complications. As reported by Loeb *et al*., the rectal bleeding rate varied between 1.3% and 45% [20, 21]. The value at our center was 1.9%. Massive rectal bleeding could be controlled by rectal balloon tamponade, endoscopic adrenaline injection or sclerotherapy, or direct vessel clipping [22–25]. Most fever patients could be cured by oral antibiotics. Septic shock need hospitalization.

With the variety of PSA screening efforts and biopsy technologies, the epidemiological and pathogenetic characteristics of PCa in Chinese Han population changed with decades. The PCDR increased over the years at our center, while the mean age of PCa patients decreased slightly, similar to the SEER database documented [26]. The mean Gleason score increased synchronization with PSA levels. However, Gleason score with PSA level lower than 4 ng/ml was abnormally higher than that of PSA level of 4.01–10 ng/ml, 10.01–20 ng/ml and 20.01–50 ng/ml. Possible cause was that most patients with PSA level lower than 4 ng/ml received biopsy due to the abnormal DRE findings or images, which usually meant a higher degree of malignancy. In addition, patients received biopsy due to clinical symptoms had usually high Gleason score. PCa was a kind of tumor with “inertia”. Some symptoms such as LUTS often meant the progressive or invasive tumor.

Anyway, PSA screening is still the most important marker to guide the prostate biopsy. The European Randomized Study of Screening for Prostate Cancer (ERSPC) recently released the 13-year follow-up results. It demonstrated the 21% reduction of PCa-specific mortality in 55- to 69-year-old male in favor of PSA screening [27]. Meanwhile, the detection rate of low-risk PCa, in the other words, clinically insignificant PCa, reached to 40% to 50% [28]. PSA screening has been carried out extensively in China. More and more patients underwent biopsy due to evaluated PSA level instead of clinical symptoms. More PCa was then diagnosed in the early stage [29]. Our data also showed that the detection rate of low-risk PCa increased from 6.5% to 15.7% after 2005.

A few limitations of our study should be noted. The total recruited patients were 3762, relatively smaller than some studies in western countries [27, 30]. In addition, it is the retrospective study. Novel prostate biopsy methods such as MP-MRI plus TRUS-guided have been performed for positioning lesions. Our study did not include these new methods and data. Our report is also lack of evidence to the aggressiveness of the cancers diagnosed using systematic 13-core transrectal prostate biopsy guided by TRUS. The lack of availability of Gleason Scores for the diagnosed cancers is another limitation. These lack information will be summarized and discussed in our further study. However, to the best of our knowledge, our study included the largest number of patients in Chinese Han population. A systematic 13-core transrectal prostate biopsy guided by TRUS is safe, effective, and economic for PCa diagnosis in developing countries like China.

## MATERIALS AND METHODS

Approval for this study was granted by the ethics committee of Nanjing Medical University (China) prior to sample collection and informed written consent was received from all patients.

From March 1999 to March 2015, 3762 patients underwent prostate biopsy at Department of Urology, The First Affiliated Hospital of Nanjing Medical University. The average age of patients was 69.1 (range 22–93) years. The inclusion criteria were as follows: PSA level of 4.0 ng/ml or greater, or PSA level less than 4.0 ng/ml with abnormal DRE findings, ultrasound or MRI images. Patients with previous prostate biopsy history, a history of PCa, and androgen ablation therapy use were excluded. All patients underwent serum tPSA and fPSA detection, DRE, and TRUS to assess the prostate volume (PV) prior to the biopsy. Finger-guided biopsy was performed on 1504 patients before December 2008. The systematic sextant biopsy was adopted before December 2006. Then extended 8-core biopsy was introduced. After January 2009, other 2258 patients underwent systematic 13-core TRUS-guided transrectal biopsy. The detailed procedure was performed as our previous study [13]. The 12 cores were evenly distributed to four vertical planes (right lateral, right medial, left medial, and left lateral) of prostate tissues, in each plane three biopsy cores were respectively located at the base, middle and apex. The extra 13th core was specifically directed towards the hypoechoic lesions on TRUS or abnormal signals on MRI. For patients with normal TRUS and MRI images, the extra 13th core was at the apex of the prostate.

Statistical analysis was performed with the Statistical Package for Social Sciences, version 19.0 (SPSS, Chicago, IL, USA) software. All data were initially tested to screen for normality and homogeneity of variance, and *T-test* was performed for the comparison of different groups. For nonparametric variables, χ^2^ test was used. Statistically significant differences were determined at *p* < 0.05.
